# Opioid use among patients with pain syndromes commonly seeking surgical consultation: A retrospective cohort

**DOI:** 10.1016/j.amsu.2021.102704

**Published:** 2021-08-11

**Authors:** Cindy Kin, Loretta Chou, Debra L. Safer, Arden Morris, Qian Ding, Amber Trickey, Sabine Girod

**Affiliations:** a Stanford University Department of Surgery, S-SPIRE, 1070 Arastradero, Palo Alto, CA, 94304, USA; b Stanford University Department of Orthopedics, 450 Broadway, Redwood City, CA, 94063, USA; c Stanford University Department of Psychiatry and Behavioral Sciences, 300 Pasteur Drive, Stanford, CA, 94305, USA

**Keywords:** Chronic pain, Opioids, Psychiatric disorders, Temporomandibular joint pain, Anorectal pain, Foot/ankle pain

## Abstract

**Background:**

Surgeons often see patients with pain to exclude organic pathology and consider surgical treatment. We examined factors associated with long-term opioid therapy among patients with foot/ankle, anorectal, and temporomandibular joint pain to aid clinical decision making.

**Methods:**

Using the IBM MarketScan® Research Database, we conducted a retrospective cohort analysis of patients aged 18–64 with a clinical encounter for foot/ankle, anorectal, or temporomandibular joint pain (January 2007–September 2015). Multivariable logistic regression was used to estimate adjusted odds ratios for factors associated with long-term opioid therapy, including age, sex, geographic region, pain condition, psychiatric diagnoses, and surgical procedures in the previous year.

**Results:**

The majority of the cohort of 1,500,392 patients were women (61%). Within the year prior to the first clinical encounter for a pain diagnosis, 14% had an encounter for a psychiatric diagnosis, and 11% had undergone a surgical procedure. Long-term opioid therapy was received by 2.7%. After multivariable adjustment, older age (age 50–64 vs. 18–29: aOR 4.47, 95% CI 4.24–4.72, p < 0.001), region (South vs. Northeast, aOR 1.76, 95% CI 1.70–1.81, p < 0.001), recent surgical procedure (aOR 1.83, 95% CI 1.78–1.87, p < 0.001), male sex (aOR 1.14, 95% CI 1.12–1.16, p < 0.001) and recent psychiatric diagnosis (aOR 2.49, 95% CI 2.43–2.54, p < 0.001) were independently associated with long-term opioid therapy.

**Conclusion:**

Among patients with foot/ankle, anorectal, or temporomandibular joint pain, the risk of long-term opioid therapy significantly increased with older age, recent psychiatric diagnoses and surgical history. Surgeons should be aware of these risk factors in order to make high quality clinical decisions in consultations with these patients.

## Highlights

• Pain diagnoses commonly seen by surgeons often have psychiatric comorbidities.
• Psychiatric disorders increase risk for long-term opioid therapy.
• Older patients are at higher risk for long-term opioid therapy.

## 1 Introduction

The lack of standardized opioid prescribing practices and diversion of unused opioid medication have been identified as major contributing factors to the deadly opioid epidemic in the U.S [[1], [2], [3]]. The medical community has recognized the critical necessity of adopting opioid-sparing methods of pain management, prevention of opioid use disorder, and the development of more personalized strategies for effective management of acute and chronic pain [[4], [5], [6]]. Beyond the necessity of managing expected postoperative pain, surgeons also interact frequently with patients with acute or chronic pain in order to rule out organic and potentially operable etiologies.

To effectively manage such pain diagnoses, surgeons must be aware of the risk factors for opioid dependence. One such risk factor is sex, as sex-related biologic differences in pain perception [7] and pharmacokinetic processes require different treatment strategies for women and men [[8], [9], [10]]. Women are more likely to experience chronic pain syndromes such as TMJ disease [11], interstitial cystitis/bladder pain syndrome [12], and fibromyalgia [13]. In addition to sex-specific biologic differences in the perception and treatment of pain, there are also sex-specific factors that mediate pain intensity and the subsequent psychological distress [14]. Overall, opioid use is also more common among women than men [15]. Psychiatric disorders also play a role in amplifying chronic pain and increasing the risk of long-term opioid use [[16], [17], [18], [19], [20]]. Patients with chronic pain syndromes are also more likely to have higher levels of depression and somatization [[21], [22], [23]].

Even when surgical consultation for acute or chronic pain reveals no discernible organic etiology of pain, patients often continue to seek care from surgeons for their symptoms, simply because there are no other physicians who feel qualified to manage the problem. The surgeon, who is the specialist of a particular anatomic site, thus bears the responsibility of offering non-operative treatment or referral to other appropriate specialists if available. Finding an effective treatment strategy may be hindered by the highly specialized nature of modern medicine, where a holistic approach to addressing pain in a discrete area may be necessary but overlooked.

To this end, we aimed to fill a critical gap in the understanding of patients with pain syndromes that overlap with surgical indications. We chose three discrete anatomic sites associated with pain syndromes for which patients often seek surgical consultation—foot/ankle, anorectal, and temporomandibular joint. Assessing the three different pain syndromes in one analysis allowed us to elucidate commonalities and differences associated with the risk of long-term opioid treatment. For each pain condition, we sought to determine how opioid treatment differs by sex, age, geographic region, psychiatric diagnoses, and recent surgery. Our hypotheses were that patients across the three different anatomic sites of pain would have similar risk factors for long-term opioid therapy, and that women would have higher risk for long-term opioid therapy due to a higher risk for certain psychiatric illnesses such as depression, anxiety, and somatization disorders.

## 2 Methods

### 2.1 Study inclusion

This retrospective cohort study was determined to be exempt from review by the institutional review board due to the deidentified nature of the data. The IBM® MarketScan® Research Database was queried for adult patients aged 18–64 with an inpatient or outpatient encounter for at least one of three chronic pain conditions from January 2007 to September 2015: 1) temporomandibular joint (TMJ) pain (International Classification of Diseases, Ninth Revision, ICD-9: 524.60, 524.61, 524.62, 524.63, 524.64, 524.69), 2) anorectal pain (ICD-9: 569.42, 564.6, 565.0), or 3) foot/ankle pain (ICD-9: 719.47). We ended the study period before implementation of International Classification of Diseases, Tenth Revision to maintain diagnosis consistency using ICD-9 codes. The index pain encounter was defined as the first encounter for the pain condition. This study has been registered in ClinicalTrials.gov (NCT04928131, https://clinicaltrials.gov/ct2/show/NCT04928131?term=NCT04928131&draw=2&rank=1).

### 2.2 Outcome measures

The primary outcome, long-term opioid therapy (LTOT), was defined as receipt of >90 days’ opioid supply within 6 months after the index pain encounter, with no supply gaps over a month (>31 days) [6]. Patients were excluded from the study if they met any of the following criteria: 1) enrollment in the database for <1 year prior or <1 year after the IPE, 2) traumatic injury in the year prior to the IPE, 3) operation ≤30 days prior to the IPE. For the analysis of the secondary outcome of new LTOT, patients were excluded if they received LTOT in the year prior to the index pain encounter. Independent variables for both outcome models included sex, age, index pain encounter setting (outpatient vs. inpatient), US Census region (Midwest, Northeast, South, West, Unknown), a surgical procedure occurring in the previous year, and encounters for psychiatric diagnoses in the previous year. Psychiatric diagnoses were identified by ICD-9 diagnosis codes (Supplement Table 1) and included: bipolar I and II, major depression, dysthymia, adjustment disorder, post-traumatic stress disorder, anxiety, obsessive compulsive disorder, personality disorders, and alcohol and drug use disorders.

**Table 1:** Descriptive comparisons by pain condition and sex.

	Foot/Ankle Pain			Anorectal pain			TMJ		
Demographics	Male 39% N = 412,179	Female 61% N = 641,133	P	Male 50% N = 127,923	Female 50% N = 125,881	P	Male 26% N = 49,682	Female 74% N = 143,851	P
Age									
Mean (SD)	45.79 (11.9)	46.72 (11.6)	<0.001^a^	43.79 (11.8)	41.95 (12.3)	<0.001^a^	42.60 (12.9)	42.67 (12.5)	0.27^a^
Median (IQR)	48 (38, 56)	49 (39, 56)	<0.001^b^	45 (35, 53)	43 (32, 52)	<0.001^b^	44 (33, 54)	44 (33, 53)	0.84^b^
**Age category, n (%)**			<0.001^c^			<0.001^c^			<0.001^c^
18-29	47,556 (42.3)	64,927 (57.7)		17,995 (42.6)	24,292 (57.4)		9687 (27.2)	25,899 (72.8)	
30-39	71,621 (42.2)	98,158 (57.8)		27,207 (48.7)	28,611 (51.3)		9908 (24.6)	30,375 (75.4)	
40-49	108,980 (39.4)	167,445 (60.6)		36,220 (52.3)	33,000 (47.7)		12,331 (24.7)	37,544 (75.3)	
50-64	184,022 (37.2)	310,603 (62.8)		46,501 (53.8)	39,978 (46.2)		17,756 (26.2)	50,033 (73.8)	
**Region**			<0.001^c^			<0.001^c^			<0.001^c^
Northeast	74,550 (39.9)	112,379 (60.1)		24,796 (51.5)	23,359 (48.5)		8812 (27.7)	22965 (72.3)	
Midwest	96,818 (39.3)	149,356 (60.7)		26,625 (50.7)	25,882 (49.3)		10,744 (26.2)	30294 (73.8)	
South	160,821 (37.9)	263,510 (62.1)		50,291 (49.6)	51,149 (50.4)		17,498 (24.1)	55003 (75.9)	
West	72,340 (41.2)	103,124 (58.8)		23,868 (51.0)	22,936 (49.0)		11,739 (26.5)	32619 (73.5)	
Unknown	7650 (37.5)	12,764 (62.5)		2343 (47.8)	2555 (52.2)		889 (23.0)	2970 (77.0)	
**Setting of index pain encounter, n (%)**			<0.001^c^			0.38^c^			0.002^c^
Outpatient	409,798 (39.1)	639,267 (60.9)		126,759 (50.4)	124,777 (49.6)		49,427 (25.7)	142,931 (74.3)	
Inpatient	2381 (56.1)	1866 (43.9)		1164 (51.3)	1104 (48.7)		255 (21.7)	920 (78.3)	
**Psych diagnosis in prior year, n (%)**									
Any psychiatric diagnosis			<0.001^c^			<0.001^c^			<0.001^c^
No	375,642 (40.9)	542,737 (59.1)		114,559 (52.3)	104,483 (47.7)		43,103 (27.5)	113,678 (72.5)	
Yes	36,537 (27.1)	98,396 (72.9)		13,364 (38.4)	21,398 (61.6)		6579 (17.9)	30,173 (82.1)	
Mood disorder			<0.001^c^			<0.001^c^			<0.001^c^
No	397,698 (40.1)	593,274 (59.9)		123,143 (51.4)	116,496 (48.6)		47,250 (26.6)	130,360 (73.4)	
Yes	14,481 (23.2)	47,859 (76.8)		4780 (33.8)	9385 (66.3)		2432 (15.3)	13,491 (84.7)	
Trauma and stressor-related disorder			<0.001^c^			<0.001^c^			<0.001^c^
No	404,892 (39.4)	622,680 (60.6)		125,469 (50.8)	121,519 (49.2)		48,571 (26.0)	138,431 (74.0)	
Yes	7287 (28.3)	18,453 (71.7)		2454 (36.0)	4362 (64.0)		1111 (17.0)	5420 (83.0)	
Anxiety/OCD/psychogenic disorder			<0.001^c^			<0.001^c^			<0.001^c^
No	392,326 (40.0)	589,128 (60.0)		119,701 (51.3)	113,457 (48.7)		45,540 (26.6)	125,552 (73.4)	
Yes	19,853 (27.6)	52,005 (72.4)		8222 (39.8)	12,424 (60.2)		4142 (18.5)	18,299 (81.5)	
Personality disorder			<0.001^c^			0.007^c^			<0.001^c^
No	411,823 (39.1)	640,278 (60.9)		127,792 (50.4)	125,705 (49.6)		49,620 (25.7)	143,524 (74.3)	
Yes	356 (29.4)	855 (70.6)		131 (42.7)	176 (57.3)		62 (15.9)	327 (84.1)	
Substance use disorder			<0.001^c^			<0.001^c^			<0.001^c^
No	410,400 (39.1)	639,996 (60.9)		127,460 (50.4)	125,653 (49.6)		49,477 (25.6)	143,532 (74.4)	
Yes	1779 (61.0)	1137 (39.0)		463 (67.0)	228 (33.0)		205 (39.1)	319 (60.9)	
**Surgical procedure in prior year**			<0.001^c^			<0.001^c^			<0.001^c^
No	377,036 (40.3)	558,634 (59.7)		114,248 (51.3)	108,386 (48.7)		45,891 (26.3)	128,319 (73.7)	
Yes	35,143 (29.9)	82,499 (70.1)		13,675 (43.9)	17,495 (56.1)		3791 (19.6)	15,532 (80.4)	

a Two-sample *t*-test.

b Wilcoxon-Mann-Whitney test.

c Chi-square Test.

### 2.3 Statistical analysis

Unadjusted analyses were performed to assess associations between patient characteristics and opioid use with chi-square tests for categorical variables, two independent sample t-tests for normally distributed continuous variables and Wilcoxon-Mann-Whitney test for nonparametric continuous variables. Frequency counts and percentages are reported for categorical variables, means (SD) and median (IQR) are presented for continuous variables. In multivariable analysis, we calculated logistic regression models to examine the independent effects of selected patient factors on LTOT and new LTOT. Multivariable models were calculated for the overall cohort (all pain conditions) as well as stratified models by pain condition. We report adjusted odds ratios (aOR), 95% confidence intervals (CI) and p-values. Statistical significance was assessed at the level of p < 0.05. All analyses were performed using SAS 9.4 (Cary, NC). This work has been reported in line with the STROCSS criteria [24].

## 3 Results

The cohort included 1,500,392 patients who had at least one inpatient or outpatient visit for the indication of foot/ankle, anorectal, and TMJ pain. The majority of the cohort were women (61%). Almost all index pain encounters were outpatient visits (99.5%). A healthcare visit for the primary indication of a psychiatric diagnosis within the previous year was observed for 14% of this cohort; women were more likely to have had such a visit compared to men (16.5% vs. 9.6%, p < 0.001). Women were also more likely to have undergone a surgical procedure within the previous year (12.7% vs. 8.9%, p < 0.001).

Characteristics of each pain cohort were detailed in Table 1. Women comprised nearly three-quarters (74%) of the TMJ group and more than half (61%) of the foot/ankle pain group, while the anorectal pain group was evenly split between women and men. Across all three pain conditions women were more likely to have encounters for psychiatric diagnoses and surgical procedures occurring in the year preceding the index pain encounter (all p < 0.001).

### 3.1 Sex differences in long-term opioid therapy

A small proportion of the overall cohort received LTOT (2.7%). When stratified by pain condition, women with TMJ and anorectal pain were more likely to receive LTOT than men, with small but statistically significant sex differences (for TMJ, 2.60% women vs. 2.23% men, p < 0.001; for anorectal, 1.90% women vs. 1.78% men, p = 0.026). Men with foot/ankle pain were more likely to receive LTOT than women (2.93% women vs. 3.02% men, p = 0.010, Table 2).

**Table 2:** Long-term opioid use by patient demographics and clinical characteristics.

	Foot/Ankle Pain		Anorectal pain		TMJ	
**Characteristics**	Long-term Opioids %	P^a^	Long-term Opioids %	P^a^	Long-term Opioids %	P^a^
**Sex**		0.010		0.026		<0.001
Female	2.93		1.90		2.60	
Male	3.02		1.78		2.23	
**Age category**		<0.001		<0.001		<0.001
18-29	0.77		0.58		0.86	
30-39	2.04		1.18		2.20	
40-49	3.01		1.97		2.82	
50-64	3.76		2.77		3.32	
**Region**		<0.001		<0.001		<0.001
Northeast	1.98		1.21		1.56	
Midwest	3.06		1.86		2.33	
South	3.31		2.08		2.89	
West	3.13		1.99		2.73	
Unknown	2.31		1.49		2.41	
**Setting of index pain encounter**		<0.001		<0.001		<0.001
Outpatient	2.94		1.77		2.47	
Inpatient	11.04		9.52		8.60	
**Psych diagnosis in prior year**						
Any psychiatric diagnosis		<0.001		<0.001		<0.001
No	2.53		1.51		1.99	
Yes	5.98		3.95		4.71	
Mood disorder		<0.001		<0.001		<0.001
No	2.72		1.66		2.22	
Yes	6.87		4.89		5.68	
Trauma and stressor-related disorder		<0.001		<0.001		<0.001
No	2.94		1.80		2.46	
Yes	3.95		3.20		3.86	
Anxiety/OCD/psychogenic disorder		<0.001		<0.001		<0.001
No	2.71		1.64		2.23	
Yes	6.48		4.06		4.66	
Personality disorder		<0.001		0.002		<0.001
No	2.96		1.84		2.50	
Yes	5.86		4.23		8.23	
Substance use disorder		<0.001		<0.001		<0.001
No	2.96		1.83		2.49	
Yes	7.41		5.64		8.02	
**Surgical procedure in prior year**		<0.001		<0.001		<0.001
No	2.68		1.53		2.24	
Yes	5.23		4.05		4.91	

a Chi-square test.

**Fig. 1:**
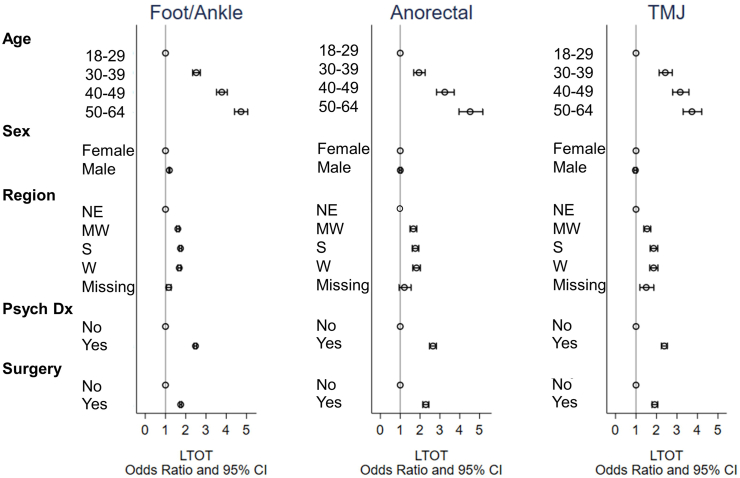
Adjusted odds ratios of characteristics associated with long-term opioid therapy among patients with foot or ankle pain, anorectal pain, or temporomandibular joint pain.

In multivariable analysis of the overall cohort, after controlling for other factors in the model, men had 14% higher odds of receiving LTOT than women (aOR 1.14, 95%CI 1.12–1.16, p < 0.001). Stratified multivariable models revealed a significantly higher odds of LTOT for men with foot/ankle pain (aOR 1.19, 95% CI 1.16–1.22, p < 0.001), but no significant sex differences in LTOT for patients with anorectal pain (aOR 1.004, 95% CI 0.95–1.07, p = 0.88) or TMJ pain (aOR 0.97, 95% CI 0.90–1.04, p = 0.37) (Fig. 1).

### 3.2 Other factors associated with long-term opioid therapy across pain conditions

Stratified analyses by pain condition subgroups indicated similar associations between LTOT and age, geographic region, encounters for psychiatric disorder in the preceding year, and surgery in the preceding year (Table 2). Older patients and those residing in the South and West were more likely to receive LTOT in all three pain condition subgroups. Patients seen for a psychiatric disorder and those who underwent a surgical procedure in the preceding year had more than twice the odds of receiving LTOT. In multivariable analyses of the overall cohort, older age, Southern region, psychiatric diagnosis and surgical procedure in the preceding year were associated with higher odds of LTOT (Table 3).

**Table 3:** Multivariable logistic regression model results for long-term opioid therapy (LTOT) and new long-term opioid therapy.

Covariates	LTOT (N = 1,500,392)		New LTOT (N = 1,475,466)	
	aOR (95% CI)	P-value	aOR (95%CI)	P-value
**Age**		<0.001		<0.001
18–29	Ref	<0.001	ref	<0.001
30–39	2.39 (2.25, 2.54)	<0.001	1.69 (1.50, 1.90)	<0.001
40–49	3.56 (3.37, 3.76)	<0.001	2.22 (1.99, 2.48)	<0.001
50–64	4.47 (4.24, 4.72)	<0.001	2.40 (2.16, 2.67)	<0.001
**Sex**				
Female	Ref		ref	
Male	1.14 (1.12, 1.16)	<0.001	1.30 (1.23, 1.36)	<0.001
**Region**		<0.001		<0.001
Northeast	Ref		ref	
Midwest	1.61 (1.55, 1.66)	<0.001	1.63 (1.50 1.78)	<0.001
South	1.76 (1.70, 1.81)	<0.001	1.83 (1.69, 1.99)	<0.001
West	1.72 (1.66, 1.78)	<0.001	1.64 (1.50, 1.80)	<0.001
Unknown	1.21 (1.11, 1.31)	<0.001	1.74 (1.45, 2.09)	<0.001
**Pain conditions**		<0.001		<0.001
Anorectal pain	ref		ref	
TMJ	1.35 (1.30, 1.41)	<0.001	1.24 (1.12, 1.37)	<0.001
Foot/Ankle Pain	1.53 (1.48, 1.58)	<0.001	1.43 (1.32, 1.54)	<0.001
Multiple pain conditions	1.27 (0.56, 2.87)	0.56	1.23 (0.17, 8.82)	0.83
**Any psychiatric diagnosis**^a^				
No	ref		ref	
Yes	2.49 (2.43, 2.54)	<0.001	2.23 (2.10, 2.36)	<0.001
**Surgical procedure in previous year**				
No	ref		ref	
Yes	1.83 (1.78, 1.87)	<0.001	1.75 (1.64, 1.87)	<0.001

aOR: Adjusted Odds Ratio.

a Psychiatric diagnoses were based on encounter diagnosis codes within 1 year prior to index pain visit.

Stratified multivariable models indicated similar results as the overall model: older age, region of residence, and recent history of a surgical procedure and recent psychiatric diagnosis were associated with higher odds of receiving LTOT (Fig. 1).

### 3.3 New long-term opioid therapy

After excluding 24,926 patients who had received LTOT during the year prior to the index pain encounter, the analysis of new LTOT was performed on a cohort of 1,475,466 opioid-naïve patients. Only 6406 patients (0.43%) received new LTOT, which was similar between women (0.41%) and men (0.47%).

The multivariable regression analysis demonstrated similar patterns of association as the univariate analysis, where men were slightly more likely to be prescribed new LTOT than women after controlling for other covariates. Patients who had encounters with a psychiatric diagnosis or a surgical procedure in the preceding year had significantly higher odds of receiving new LTOT regimen after adjustment for covariates (psychiatric diagnosis: aOR, 2.23, 95% CI 2.10–2.36, p < 0.001; surgical procedure: aOR, 1.75, 95% CI 1.64–1.87, p < 0.001, Table 3). In stratified multivariable analysis, there was a small but significantly higher likelihood for men with foot/ankle pain to receive new LTOT (aOR 1.37, 95% CI 1.29–1.45, p < 0.001). Older age, geographic region, an encounter with psychiatric diagnosis and a surgical procedure in the preceding year were all associated with higher rates of new LTOT (all p < 0.001, Supplemental Table II).

## 4 Discussion

This study examined the risk factors for long-term opioid use in patients presenting with new diagnoses of foot/ankle, anorectal, and TMJ pain. These diagnoses may have potential surgical treatments, but may also be caused by conditions that cannot be corrected surgically. We found that recent psychiatric diagnoses, recent surgery, residence outside the Northeast region and older age were independently associated with long-term opioid use in all three pain conditions. Contrary to our hypothesis that women would be at greater risk for long-term opioid therapy, our analyses found no sex differences in LTOT for patients with TMJ or anorectal pain, and 16% lower odds of LTOT for women with foot/ankle pain. These risk factors and patterns were reflected in the analysis of new LTOT as well.

Notably, patients seen for psychiatric diagnoses in the preceding year had nearly 2.5 times the odds of requiring LTOT as those without those diagnoses. Mood disorders accountd for over 40% of these psychiatric diagnoses, with anxiety, OCD, and psychogenic disorders accounting for the majority of the remainder. Our findings are supported by studies that have found associations between psychiatric disorders and initiation and use of prescribed opioids [[17], [18], [19]]. TMJ pain has long been associated with depression and somatization disorders, and there is evidence of a high prevalence of anxiety, depression and neuroticism in patients with foot and ankle pain; however this association has not been noted for patients with anorectal pain [21,25,26]. Our analysis on TMJ and foot/ankle pain was consistent with these prior findings, but we also found that 14% of patients with anorectal pain had recent encounters for psychiatric disorders, which has not been previously described.

Consistent with prior epidemiological reports of chronic pain conditions, there was a high proportion of women in our study population [27]. While the majority of patients with TMJ pain (74%) and anorectal pain (61%) were women, the distribution in anorectal pain was even. Across all three pain condition subgroups, the proportion who had a recent psychiatric diagnosis was higher in women, as was the proportion who had a recent operation.

This study has several limitations – mostly due to the inherent characteristics of retrospective analysis and claims data, which preclude the ability to make causal interpretations. Additionally, we were unable to include patients 65 and older since this database was a private insurance claims and thus would not have included full claims information on the older beneficiaries of Medicare.

## 5 Conclusions

In a health care system that is increasingly specialized and siloed, our findings demonstrate the importance of patient-centered and multidisciplinary approaches to treat highly-specialized pain syndromes. It is important for surgeons to recognize the risks associated with initiation of chronic opioid use in this patient population, as comorbid psychiatric disorders are likely to complicate the management of pain. Further investigation to elucidate connections between psychiatric diagnoses and pain is critical to understand the pathophysiology of these disorders and to develop effective treatment strategies. Understanding the risk factors for LTOT in patients presenting for evaluation of pain symptoms that may or may not be surgically treated will inform treatment decision-making for surgeons and other providers who see these patients.

## Declaration of competing interest

No conflicts of interest to declare.

## Trial registry number

1. Name of the registry: clinicaltrials.gov
2. Unique Identifying number or registration ID: NCT04928131
3. Hyperlink to your specific registration (must be publicly accessible and will be checked): https://clinicaltrials.gov/ct2/show/NCT04928131?term=NCT04928131&draw=2&rank=1
