# Supplementary material for: Opioid use among patients with pain syndromes commonly seeking surgical consultation: A retrospective cohort
Source: Ann Med Surg (Lond). 2021 Aug 11;69:102704. doi: 10.1016/j.amsu.2021.102704 (PMC8384768; doi:10.1016/j.amsu.2021.102704)
Supplement: Multimedia component 2 [file mmc2.docx]

**Supplemental**

**Supplemental Table I: Psychiatric diagnosis codes and classification categories utilized in analyses.**

| **Category** | **Description** | **ICD-9 diagnosis codes** |
| --- | --- | --- |
| Mood disorders | Bipolar I and II disorders | 296.0, 296.1, 296.4, 296.5, 296.6, 296.7, 296.80, 296.89 |
|  | Major Depressive Disorder | 296.2 |
|  | Major Depressive Disorder | 296.3 |
|  | Dysthymic disorder | 300.4 |
|  | Depressive disorder, not elsewhere classified | 311 |
|  | Depression | V79.0 |
| Trauma and stressor-related disorders | Adjustment disorder with anxiety | 309.24, 309.28 |
|  | Adjustment disorder with depression | 309.0, 309.1 |
|  | Post-traumatic stress disorder | 309.81 |
| Anxiety, obsessive-compulsive,  psychogenic, and somatoform disorders | Anxiety | 300.00,300.01, 300.02, 300.09 |
|  | Obsessive compulsive disorders | 300.3 |
|  | Psychogenic disorders, hypochondriasis, somatoform | 306.xx, 300.7, 300.8x |
|  | Psychosexual dysfunction | 302.7x, 799.81 |
|  | Sleep syndromes not due to substance or known physiologic condition | 307.4x |
| Personality disorders |  | 301, 301.xx |
| Substance-related disorders | Alcohol use/dependence/abuse | 303, 303.9, 305.0 |
|  | Drug use/dependence/abuse of sedatives, anxiolytic, cocaine, cannabis, amphetamines, hallucinogens | 304.1, 304.2, 304.3, 304.4, 304.5, 304.6, 304.8, 304.9, 305.2, 305.3, 305.4, 305.6, 305.7, 305.8, 305.9 |
|  | Alcohol-induced mood, anxiety, sexual, or sleep disorders | 291.82, 291.89 |
|  | Drug-induced mood, anxiety, sexual, or sleep disorders | 292.84, 292.85, 292.89 |

**Supplemental Table II: Multivariable models predicting new long-term opioid therapy, stratified by pain condition.**

|  | **Foot/Ankle Pain** |  | **Anorectal pain** |  | **TMJ** |  |
| --- | --- | --- | --- | --- | --- | --- |
| **Covariates** | **aOR (95%CI)** | **P-value** | **aOR (95%CI)** | **P-value** | **aOR (95%CI)** | **P-value** |
| **Age** |  | <0.001 |  | <0.001 |  | <0.001 |
| 18-29 | ref |  | ref |  | ref |  |
| 30-39 | 1.89 (1.62, 2.19) | <0.001 | 1.07 (0.80, 1.44) | 0.65 | 1.69 (1.28, 2.23) | <0.001 |
| 40-49 | 2.39 (2.078 2.76) | <0.001 | 1.83 (1.41, 2.38) | <0.001 | 2.11 (1.63, 2.74) | <0.001 |
| 50-64 | 2.59 (2.26, 2.97) | <0.001 | 2.45 (1.91, 3.14) | <0.001 | 1.84 (1.42, 2.37) | <0.001 |
| **Sex** |  |  |  |  |  |  |
| Female | ref |  | ref |  | ref |  |
| Male | 1.37 (1.29, 1.45) | <0.001 | 1.09 (0.95, 1.25) | 0.22 | 1.02 (0.86, 1.21) | 0.86 |
| **Region** |  | <0.001 |  | <0.001 |  | <0.001 |
| Northeast | ref |  | ref |  | ref |  |
| Midwest | 1.64 (1.49, 1.82) | <0.001 | 1.54 (1.21, 1.96) | <0.001 | 1.63 (1.22, 2.17) | <0.001 |
| South | 1.84 (1.68, 2.02) | <0.001 | 1.58 (1.27, 1.96) | <0.001 | 2.14 (1.65, 2.77) | <.0001 |
| West | 1.57 (1.41, 1.75) | <0.001 | 1.74 (1.37, 2.22) | <0.001 | 2.024 (1.53, 2.67) | <.0001 |
| Unknown | 1.86 (1.52, 2.28) | <0.001 | 1.08 (0.59, 1.96) | 0.81 | 1.75 (0.999, 3.06) | 0.051 |
| **Any psychiatric diagnosis** |  |  |  |  |  |  |
| No | ref |  | ref |  | ref |  |
| Yes | 2.25 (2.11, 2.41) | <0.001 | 2.09 (1.78, 2.46) | <0.001 | 2.21 (1.89, 2.57) | <0.001 |
| **Surgical Procedure in Prior Year** |  |  |  |  |  |  |
| No | ref |  | ref |  | ref |  |
| Yes | 1.64 (1.52, 1.77) | <0.001 | 2.32 (1.98, 2.73) | <0.001 | 1.88 (1.55, 2.27) | <0.001 |

aOR: adjusted odds ratio
